# Laser-Free Photosensitive Systems in Cancer Therapy: A Comprehensive Review

**DOI:** 10.3390/ijms26041437

**Published:** 2025-02-08

**Authors:** Ruixue Jia, Shuyun Zhang, Jicheng Zhang, Yi Li

**Affiliations:** Academy of Pharmacy, Xian-Jiaotong Liverpool University, Suzhou 215000, China; jrx127168@163.com (R.J.); shuyun.zhang23@student.xjtlu.edu.cn (S.Z.); jicheng.zhang23@student.xjtlu.edu.cn (J.Z.)

**Keywords:** photodynamic therapy (PDT), resonance energy transfer (RET), chemically induced electron exchange luminescence (CIEEL), Cherenkov radiation energy transfer (CRET), laser-free system

## Abstract

Photodynamic therapy (PDT) involves the use of photosensitizers (PSs) that, upon activation by specific wavelengths of light, generate reactive oxygen species (ROS), including singlet oxygen (^1^O_2_) and hydroxyl radicals (·OH), within the targeted tissue, typically tumor cells. The generated ROS induces cellular damage, disrupts cellular processes, and ultimately leads to apoptosis or necrosis of the tumor cells. However, the clinical application of PDT is significantly hindered by the limited tissue penetration ability of light. To address this limitation, laser-free self-luminescent photosensitive systems have emerged as potential solutions for achieving deep-tissue PDT and imaging. This review provides a comprehensive analysis of various laser-independent photosensitive systems, with a particular emphasis on those based on resonance energy transfer (RET), chemically induced electron exchange luminescence (CIEEL), and Cherenkov radiation energy transfer (CRET). The aim is to offer a theoretical framework for the development of novel photodynamic systems and to reassess the application potential of certain previously overlooked photosensitizers (PSs).

## 1. Introduction

Tumors have always been a significant threat to human health. According to the study released by International Agency for Research on Cancer (IARC) in 2024, one in five people will develop cancer in their lifetime, and the global death toll from cancer was projected to reach 9.7 million in 2022 [1]. Photodynamic therapy (PDT), as an emerging non-invasive anti-tumor treatment, is increasingly used in various types of tumors. The photosensitizers (PSs) can generate reactive oxygen species (ROS) when exposed to light of a specific wavelength [2,3]. This induces an imbalance in the tumor cell redox microenvironment, causing an excessive accumulation of ROS that exceeds the toxicity threshold, and ultimately triggers various programmed cell deaths [4,5,6]. Due to the high precision of external light irradiation, photosensitizers, although typically administered systemically, only exert biological activity in the irradiated area. As a result, PDT is considered to have more potential in tumor treatment compared to traditional radiotherapy and chemotherapy.

To fully exploit the potential of photodynamic therapy (PDT), three essential conditions must be met: the presence of light, photosensitizers, and oxygen, as outlined in the fundamental mechanism of PDT [3,7,8]. PDT consists of two sequential processes: Initially, PSs in their ground state accumulate in tumor tissue. Upon absorbing photons energy, these PSs transition to an excited singlet state. Through intersystem crossing (ISC), the unstable singlet-state PSs convert to a triplet state, where PSs generate various bio-toxic ROS through electron or energy transfer mechanisms [9,10]. There are two types of photosensitizers: Type II PSs rely on tissue oxygen and convert triplet oxygen into singlet oxygen (^1^O_2_) through energy transfer, while type I PSs are less reliant on oxygen and can generate diverse ROS through electron transfer. Regardless of the type of photosensitizer, illumination from a light within the excitation band is necessary [3,8,11]. The excitation light sources for commonly used photosensitizers, such as 5-ALA, Chlorin e6, phthalocyanines, etc., are primarily blue light and near-infrared (NIR) light. The near-infrared light, which offers the strongest tissue penetration, typically penetrates tissues by less than 1 mm, while the blue light exhibits even weaker penetration [12]. Extracorporeal light source has been a significant advantage of PDT, allowing for spatiotemporally controlled activation of PSs. However, as PDT has advanced, in vitro light sources have become a limitation in clinical application due to their insufficient tissue penetration.

**Figure 1:**
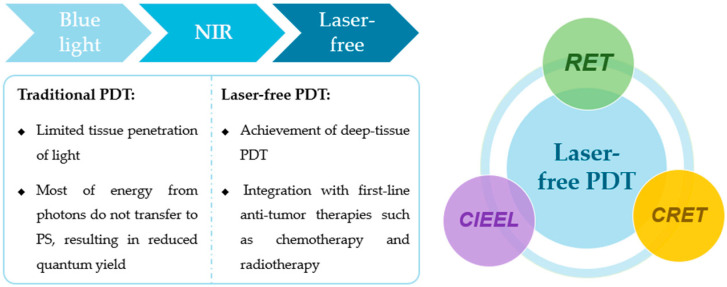
Schematic representation of the PDT evolution, the main categories of laser-free PDT and their differences from traditional PDT.

In recent years, there has been a surge in research aimed at enhancing the photosensitive system to overcome the limitations of external light sources and enable in vivo self-luminescence for PDT. These laser-free PDT systems herald a renaissance for many previously overlooked blue light-dependent photosensitizers, offering the potential for effective deep tissue treatment and imaging (Figure 1). This article reviews the latest advancements in laser-free PDT systems, with a special emphasis on resonant energy transfer (RET), chemically induced electron exchange luminescence (CIEEL), and Cherenkov radiation energy transfer (CRET). It aims to provide a theoretical foundation for the development of novel laser-free PDT systems, thereby expanding the application scope of PDT and offering new insights into various combination therapies for tumors.

## 2. Laser-Free PDT Based on Resonance Energy Transfer (RET)

**Figure 2:**
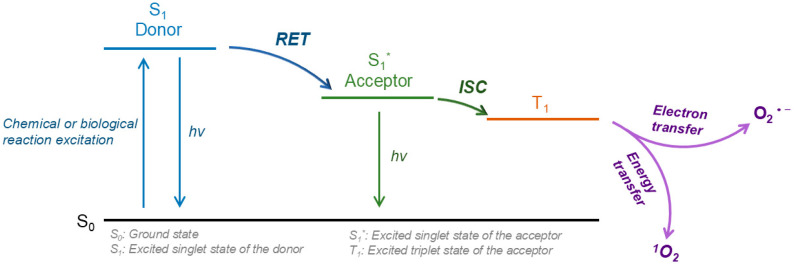
The general process of the RET-induced PDT.

The most widely studied laser-free PDT system is based on Resonance Energy Transfer (RET). RET arises from the dipole–dipole interaction between two closely spaced fluorescent molecules: the donor molecule and the acceptor molecule. When the donor is excited, energy is transferred to receptors located within a critical radius, leading to a decrease in donor fluorescence and an increase in receptor fluorescence [13,14]. The stimulated receptors, i.e., the PSs, can generate different types of ROS, actually exert bioactivity through electron transfer (type I PS) or energy transfer (type II PS) (Figure 2). The selection of donor and acceptor molecules must meet two conditions: (1) the emission spectrum of the donor overlaps with the excitation spectrum of the acceptor [15] and (2) the distance between the donor and acceptor is less than the critical radius (1–10 nm) [16]. The alignment of the energy levels of the donor and acceptor is also a crucial factor that can greatly influence the efficiency of RET.

Currently, research on RET is mainly categorized into three key areas: Chemiluminescent Resonance Energy Transfer (sometimes abbreviated as CRET, though, in this review, CRET specifically refers to Cherenkov Radiation Energy Transfer), Bioluminescence Resonance Energy Transfer (BRET), and Fluorescence Resonance Energy Transfer (FRET). However, since FRET is limited by the need for excitation light to excite donor molecules, this review will focus on chemiluminescence and bioluminescence RET rather than FRET. Given the highly similar mechanisms underlying both chemiluminescence and bioluminescence RET, which often lead to confusion in some research, this article primarily categorizes various RET systems based on the donor molecules involved, without making a detailed distinction between the two.

### 2.1. Luminol/H_2_O_2_ Donor Systems

**Figure 3:**
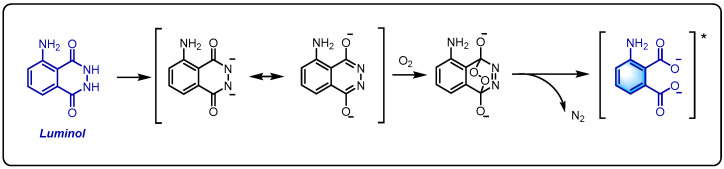
General mechanism of luminol-induced chemiluminescence [24].

So far, the most extensively studied donor luminescence primarily uses blue light, with luminol being one of the most widely used substances. Luminol is a classic chemiluminescent reagent that, when oxidized by H_2_O_2_ in the presence of certain catalysts (such as horseradish peroxidase (HRP), Fe^2^+, or Cu^2^+), produces an excited-state aminophosphate that emits blue light (around 425 nm) for approximately 30 min (Figure 3) [17,18]. Due to the overexpression of H_2_O_2_ in the tumor microenvironment (TME) [19,20,21], luminol has always been regarded as an excellent RET donor molecule. In 2006, Huang et al. first proposed the concept of chemiluminescence RET and achieved energy transfer between luminol and quantum dots through the luminol/H_2_O_2_ chemiluminescence reaction catalyzed by HRP [22]. However, H_2_O_2_ also reacts with PSs (acceptor), leading to irreversible photobleaching and a subsequent reduction in the chemiluminescence efficiency (CL). Consequently, the CL collection efficiency in this system is low, with a chemiluminescence RET ratio of only about 0.3. In 2012, Chen et al. first proposed the concept of in situ PDT using Fe^2+^, luminol, and 5-aminolevulinic acid (5-ALA) [23]. Since 5-ALA facilitated the more rapid synthesis of protoporphyrin IX (PpIX) (the actually effective PS) in normal cells compared to tumor cells, the excitation of luminol was timed to coincide with this synthetic window, thereby safeguarding normal cells. Despite the luminol’s light dose being significantly lower than that of direct light irradiation, this combined approach still demonstrates a notable capacity to trigger cell necrosis and apoptosis in vitro, hinting at promising potential for the further advancement of the luminol donor.

**Figure 4:**
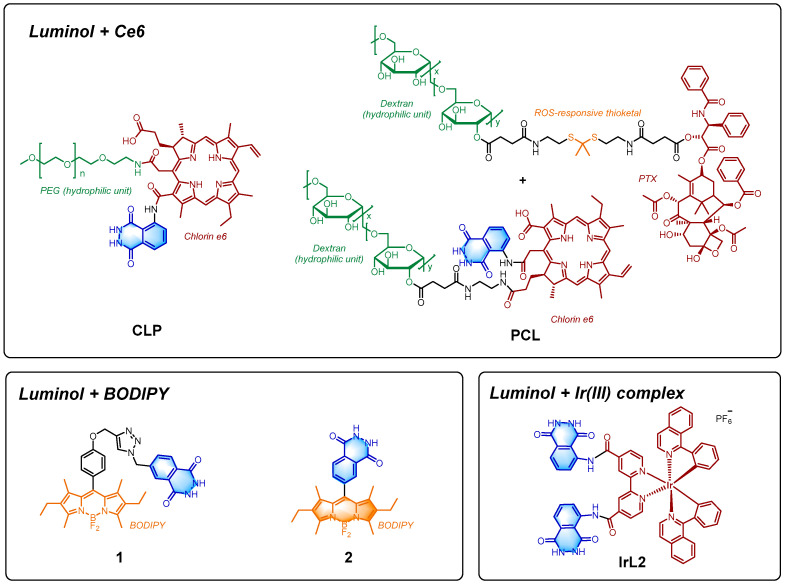
Structures of chemiluminescence RET nanodrugs based on luminol/H_2_O_2_.

In recent years, the application of luminol reaction has expanded, and the above-mentioned problems have significantly been addressed. For example, Xu et al. designed a nanoparticle called CLP, containing Chlorin e6 (Ce6), luminol, and polyethylene glycol (PEG) units (Figure 4), for inflammation imaging and tumor treatment [25]. The CL reaction of nanoparticles is driven by the elevated hydrogen peroxide levels in the TME. Since luminol and Ce6 are covalently linked, the distance between them is optimized for efficient energy transfer. While the system yields promising results in imaging inflammatory tissues, its effectiveness in tumor treatment is less satisfactory. This limitation is not only due to CL efficiency issues and the absence of catalyst but also largely stems from factors such as the structural design of nanoparticles, drug dosage, and the ability of Ce6 to generate singlet oxygen. To enhance the efficiency of CL, the types of catalyst that facilitate the oxidation of luminol are crucial. Weng et al. innovatively combined the hemin/G-quadruplex DNAzyme-loaded UiO-66 nanoplatform with chemiluminescence RET-based photodynamic therapy (PDT), creating an integrated nanoplatform for both targeted therapy and imaging [26]. In order to address the issue of insufficient treatment effectiveness, some researchers have also explored combination therapy. For example, Ding et al. synthesized multi-component PCL-NPs (Figure 4) based on pectin, incorporating a ROS-activable thioketal-based paclitaxel (PTX) prodrug along with luminol/Ce6 [27]. ROS generated from chemiluminescence RET-mediated PDT primarily serves to release PTX and achieve the combination therapy of PDT and chemotherapy, directly mediating cell death.

In addition to Ce6, boron-dipyrromethene (BODIPY) is another widely used class of photosensitizers (PS). Degirmenci et al. developed two heavy atom-free luminol-BODIPY small molecule drugs (Figure 4, compounds **1** and **2**), which are designed to enhance the yield of triplet oxygen by enhancing the intersystem crossing (ISC) process [28]. The distance between the luminol unit and BODIPY units in compounds **1** and **2** differs, resulting in RET efficiencies of 0.53 and 0.99, respectively. Between these two, compound **2**, which is activated exclusively by H_2_O_2_, demonstrates high selectivity. However, both compounds exhibited low activity in laser-free in vitro experiments, likely due to the possible absence of catalyst, although they did show cytotoxicity upon red light excitation. In 2024, Wu et al. employed a similar approach to activate hybrid protein oxygen nanocarrier coated graphene quantum dots through chemiluminescence RET, using luminol as the donor. This strategy resulted in an significant in vitro anti-tumor activity at 16 μg/mL of the nanosystem [29]. Since luminol was placed on a separate nanoparticle, its potential toxic effects may be mitigated through controlled, separate administration.

The aforementioned photosensitizers (PSs) are both type II PSs, with their ability to generate singlet oxygen largely dependent on the oxygen content in tumor tissue. In contrast, the Ir(III)-based nanoplatform IrL2H developed by Liu et al. is a type I PS, capable of generating various ROS such as superoxide radicals (O2^·−^). IrL2H liposomes contain Ir(III) complexes covalently linked to two molecules of luminol (IrL2, Figure 4), with hemoglobin (Hb) serving as a catalyst for the chemiluminescence reaction. IrL2H is effective in hypoxic tumor tissues, producing enhanced ROS, and demonstrating strong activity in both in vivo and in vitro experiments [30].

The blue light emitted by luminol/H_2_O_2_ chemiluminescence (CL) reaction aligns with excitation wavelengths of various PS receptors, making it highly applicable in PDT. However, several challenges remain that restrain the clinical applicability of luminol in certain contexts: (1) Luminol is highly susceptible to oxidation by hydrogen peroxide (H_2_O_2_) in alkaline environments (pH ≥ 9), but the tumor microenvironment (TME) typically has a much lower pH, which results in reduced chemiluminescence (CL) efficiency in the absence of effective catalysts. This discrepancy in pH can hinder the compound’s performance under physiological conditions [28,29]. (2) There is evidence suggesting that luminol may have potential genotoxicity, raising concerns about its safety profile, especially with prolonged exposure or in high concentrations [30]. (3) Luminol has a high affinity for proteins, which could lead to non-specific binding and interfere with its intended actions, potentially affecting its overall therapeutic efficacy and bioavailability [31]. These issues need to be addressed for luminol to be considered a viable option for clinical use. Currently, its use is largely limited to tumor imaging. To unlock luminol’s full therapeutic potential, the development of advanced drug delivery systems and more efficient resonance energy transfer (RET) pathways may be necessary. The ultimate goal would be to achieve cytotoxicity primarily through photodynamic therapy (PDT), rather than relying solely on the drug molecules themselves.

### 2.2. Luciferase-Catalyzed Donor Systems

**Figure 5:**
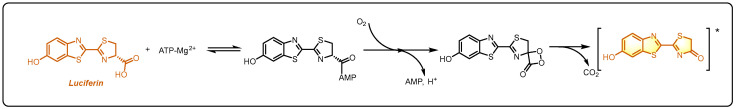
General mechanism of luciferase-catalyzed bioluminescence [24].

Alongside luminol, the luciferase-catalyzed luciferin system is another important example of resonance energy transfer (RET). Luciferase is a general term for enzymes that produce biological fluorescence. Currently, the research mainly focuses on firefly luciferase (FL), which catalyzes the oxidative decarboxylation of D-luciferin in the presence of Mg^2+^, ATP, and oxygen, emitting yellowish-green visible light (550–580 nm) (Figure 5). Since its first isolation in 1956, FL has received widespread attention across various fields. The reaction catalyzed by luciferases is considered a key representative of bioluminescence energy resonance transfer (BRET). FL-catalyzed D-luciferin was used as the donor to activate various PSs, such as Rose Bengal [31], hypericin [32], m-THPC (Temoporfin) [33], and quantum dots [34].

**Scheme 1:**
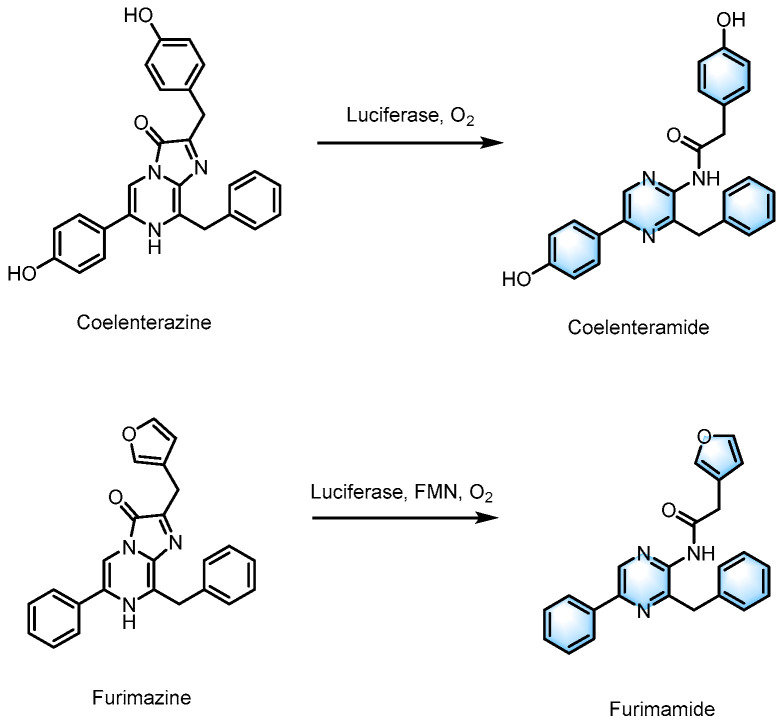
Structures of luciferase substrates and their catalytic products.

In addition to traditional FL/D-luciferin, various novel luciferase enzymes and substrates have also been developed. Coelenterazine (CTZ), a luciferin derived from marine organisms, can be oxidized and emit light at 400–480 nm in the presence of luciferase with only oxygen as a cofactor (Scheme 1) [35,36]. The RLuc8-Ce6 conjugates, developed by Yan and colleagues, activate the substrate methoxy e-coelenterazine (a CTZ analog) and stimulate Ce6 to achieve PDT through the *Renilla renormalizis* Luciferase 8 (RLuc8) protein [37]. As a natural low-toxicity compound, CTZ can be administered via direct intravenous injection in in vivo experiments. In vitro studies have demonstrated that the conjugates exhibit a significantly lower IC_50_ in 4T1 cells and MDA-MB-231 cells compared to laser-induced PDT. Additionally, the activation rate of this conjugate, defined as the ratio of photons transferred to activate Ce6 to the total photons generated by bioluminescence, can reach up to 80%. This conjugate has also shown a strong ability to produce ROS in in vivo and in vitro experiments, leading to complete tumor regression in the early three negative breast cancer (TNBC) tumor modeling.

Although small molecule fluorescent substrates are generally regarded as having low toxicity, they may still pose other risks in therapeutic applications. In contrast, more stable and less toxic photosensitive proteins offer the potential for expanding the application of BRET-PDT. In 2022, Shramova’s team proposed a gen-encoded PDT platform that utilizes genetically engineered NanoLuc luciferase to catalyze furimazine as the donor and phototoxic flavoprotein miniSOG as the PS while generating multiple ROS through type I and II photoreactions [38]. However, NanoLuc catalysis requires flavin mononucleotide (FMN) as a cofactor, so additional riboflavin mononucleotide needs to be supplied to enhance the endogenous concentration of FMN. In 2023, the same BL reaction and the SOPP3 protein were employed by Shramova’s team to achieve a higher ^1^O_2_ yield and higher photoinduced phototoxicity [39]. Disappointedly, in the absence of external laser irradiation, the PDT system lacked the targeting capability typically associated with light-activated therapies. In 2024, this limitation was addressed by incorporating a targeting protein module, DARPin, into the NanoLuc-SOPP3 BRET platform. Such modification enabled successful targeting of HER2-positive cells in both in vitro 3D spheroid models and in vivo experiments [40].

Ideally, the emission light of the donor and the excitation light of the acceptor should overlap as much as possible, but not all donor/acceptor pairs can perfectly meet this spectral overlap requirement. Therefore, a two-stage relay energy transfer process, “donor–intermediate–acceptor” may be more suitable for achieving efficient RET. Foscan^®^ (m-THPC) is a commercially available photosensitizer with an excitation wavelength of 652 nm. The emission wavelength of coelenterazine catalyzed by luciferase (RLuc8) ranges between 390 and 600 nm, which does not effectively overlap with the excitation wavelength, thus hindering efficient RET. Therefore, Hsu et al. incorporated quantum dots (QDs-655, *λ*_abs_ = 600–710 nm) into their BRET-based nanoplatform, enabling effective activation by BRET to meet the requirements of Foscan^®^ [41]. Through the energy transfer of quantum dots, the BRET ratio can reach 0.92, and in vivo experiments have shown significant inhibition of tumor tissue growth.

The luciferase-based BRET system requires the coexistence of substrates, luciferase, and its cofactor to effectively transfer energy to the PS acceptor, a complex process that may introduce unintended errors in tissues. Additionally, the toxicity associated with systemic administration of these substrates and cofactors presents a significant challenge to its practical application. Furthermore, the immunogenicity of luciferase, an exogenous protein, upon release in vivo remains unclear. Therefore, future research may focus on developing fluorescent enzymes that function independently of cofactors and novel fluorescent substrates with reduced toxicity.

## 3. Chemically Initiated Electron Exchange Luminescence (CIEEL)

**Scheme 2:**
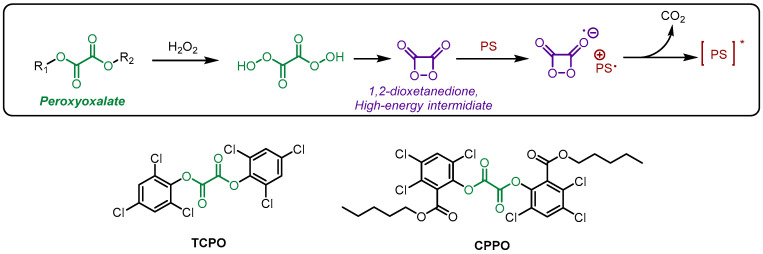
General mechanism of peroxyoxalate-induced chemiluminescence RET.

Another form of chemiluminescence (CL) besides RET is chemically initiated electron exchange luminescence (CIEEL). At present, the application of CIEEL is mainly focused on Peroxyoxalate (PO) systems. Luminol exhibits unstable CL efficiency and potential toxicity, while the luciferase system appears too complex for use in biological tissues. In contrast, Peroxyoxalate Chemiluminescence (POCL) is a promising alternative to luminol and luciferase. PO does not require additional catalysts and can react in a H_2_O_2_ environment to produce 1,2-dioxetanedione, a high-energy intermediate (HEI). This HEI can further interact with an activator, such as a PS in PDT systems, to excite the PS through CIEEL (Scheme 2) [42,43]. Unlike the Förster energy transfer process in RET, the Dexter mechanism governs the energy transfer in CIEEL. Förster energy transfer relies on dipole–dipole interactions and can occur over longer distances, while Dexter energy transfer is a spin-conserving process that depends on electron interactions and requires the overlap of electron clouds [44,45], necessitating a shorter distance between the energy donor and acceptor. Therefore, CIEEL does not require spectral overlap between the donor and acceptor; instead, it primarily depends on the proximity of the HEI and activator molecules [46]. CIEEL possesses the capability to emit light of multiple colors from a single donor, rendering it a more versatile and widely used technique in imaging applications.

TCPO, a classic molecule in peroxyoxalate systems (Scheme 2), was employed by Liang et al. to activate Ce6. As a type II photosensitizer (PS), Ce6 generates reactive oxygen species (ROS) in an oxygen-dependent manner. To address this, perfluorohexane (PFC) was incorporated into the nanoparticles to serve as an oxygen carrier [47]. Given the close relationship between POCL efficiency and H_2_O_2_ levels in tumor tissue, increasing the endogenous H_2_O_2_ levels clearly has the potential to enhance POCL efficiency. Yu et al. employed glucose oxidase (GOx) to catalyze the conversion of glucose to H_2_O_2_ and induce glucose starvation in the CPPO-Ce6 nanosystem, a strategy similar to that of Liang et al. [48]. In in vivo experiments, this strategy showed good efficacy against both primary and metastasis tumors. Similarly, Mao et al. employed a comparable approach to develop an integrated nanoprobe for treatment and imaging [49]. They selected TBD, a PS with far red/near-infrared (FR/NIR) emission and high singlet oxygen yield, as the receptor for the nanoprobes. Notably, the alignment of HOMO of TBD with the LUMO energy levels of 1,2-dioxetanedione intermediate significantly enhances energy transfer, thus improving the efficiency of PS excitation [50]. Likewise, Liu et al. incorporated cinnamaldehyde (CA) to upregulate intramolecular NADPH levels, which indirectly elevates the concentration of H_2_O_2_ [51].

**Figure 6:**
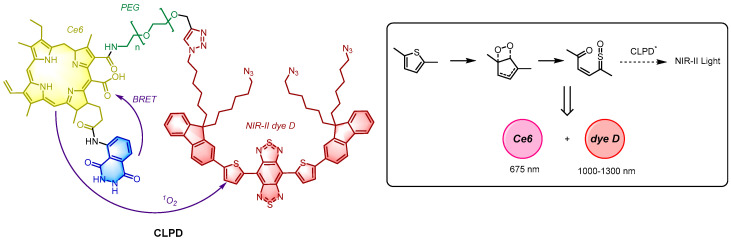
Structure and general mechanism of CLPD.

In contrast, a more advanced approach was adopted by Yuan et al. through a luminol-Ce6-NIR-II dye D nanosystem, referred to as CLPD (Figure 6). In this system, luminol is oxidated by H_2_O_2_ and excess myeloperoxidase (MPO) present in tumor tissue, which subsequently activates Ce6 via Förster resonance energy transfer (RET). The ^1^O_2_ generated by activated Ce6 then oxidizes the S-C bond of thiophene in dye D through π^2^-π^2^ cycloaddition, forming a thiophene-dioxetane intermediate. Although the absorption band of dye D overlaps with the emission band of Ce6, its excitation is not driven by RET but rather by CIEEL following ^1^O_2_ oxidation. In both in vitro and in vivo experiments, the CLPD system demonstrated a significant PDT therapeutic effect and enabled deep tissue imaging of inflammatory regions [52]. The CIEEL process observed in CLPD is independent of peroxyoxalate compounds and holds substantial potential for the development of novel laser-free CIEEL-mediated photosensitive systems.

**Figure 7:**
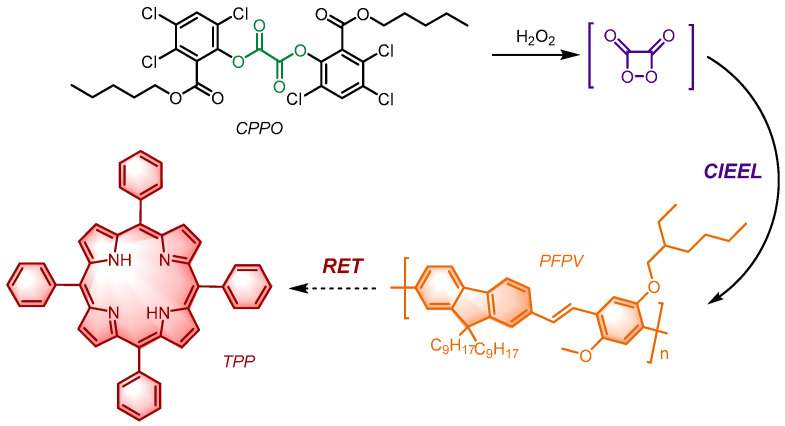
Schematic representation of the POCL system, including chemical structures of its constituents, the underlying mechanism of CIEEL, and chemiluminescence RET.

The combination of CIEEL and RET-mediated luminescence represents another promising approach. The nanoparticle POCL (Figure 7) developed by Wu et al. incorporates CPPO and the first-generation photosensitizer tetraphenylporphyrin (TPP), alongside PFPV, as a chemiluminescence donor to enhance energy transfer efficiency [53]. Notably, the emission wavelength of PFBT can overlap with the Q-band (weak absorption band generated by the n–π transition of porphyrin compounds) of TPP; however, due to the large difference in energy levels between its HOMO and LUMO of HEI in CIEEL, it is not an effective intermediate for energy transfer. On the other hand, PFPV satisfies the energy transfer requirements of the previous stage and can also align with the Q-band absorbance wavelength of TPP, thereby significantly improving the quantum yield.

**Figure 8:**
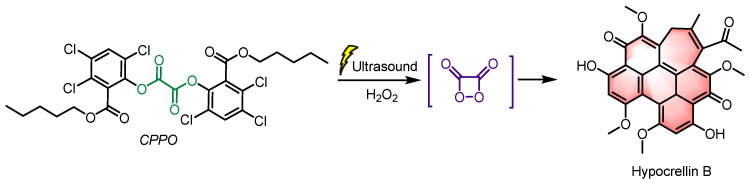
Schematic representation of the UECL-mediated structure of hypocrellin B.

Additionally, ultrasound enhanced chemiluminescence (UECL) is gaining increasing attention, as ultrasound can enhance the intensity of CL [54]. Ding et al. developed a UECL PDT system, using CPPO as the donor and hypocrellin B (Figure 8, a type II PS from Chinese natural herbs with a high ^1^O_2_ yield) as the acceptor [55]. This nanoparticle was introduced into A549 cells cultured in vitro. Without ultrasound, there was minimal ROS production; however, after ultrasound treatment (1 W/cm^−2^, 1 MHz, 50% cycle, 30 s), significant death was observed. With the development of sonodynamic therapy, an increasing number of PSs are being recognized for their sono-sensitive characteristics. This nanosystem, with its UECL-RET mechanism, can potentially exert a synergistic effect in photo-sonosensitizers, creating an integrated ultrasound-mediated platform for photo-sonodynamic therapy.

The primary constraint of the PO systems, as revealed by their applications, lies in the concentrations of H_2_O_2_ and oxygen. Nevertheless, this challenge can be addressed through established strategies, such as oxygen self-supply mechanisms and methods to enhance H_2_O_2_ levels. Additionally, UECL shows significant potential when combined with sonodynamic therapy, further broadening the scope of the chemiluminescence applications.

## 4. Cherenkov Radiation Energy Transfer (CRET)-Induced PDT

When a charged particle (usually an electron) moves through medium at a speed exceeding the speed of light, it disturbs the electromagnetic field in the medium. This disturbance generates a shockwave-like electromagnetic field structure that trails behind the particles. This shockwave propagates at the speed of light in the medium and emits electromagnetic radiation, a phenomenon known as Cherenkov radiation [56,57,58]. Cherenkov radiation is produced by the decay of radionuclides, such as ^82^Gb, ^89^Zr, and ^198^Au, which emit β^+^ particles, and ^90^Y, ^131^I, and ^177^Lu, which emit β^−^ particles. These high-speed particles induce local polarization along their direction of motion, generating detectable optical signals known as Cherenkov luminescence, as the medium returns to equilibrium. The spectrum of Cherenkov luminescence is continuous (200–1000 nm), with the highest intensities in the high-frequency ultraviolet (UV) and blue light range (200–400 nm). This light is sufficiently intense to excite photosensitizers (PSs) [59,60,61].

**Figure 9:**
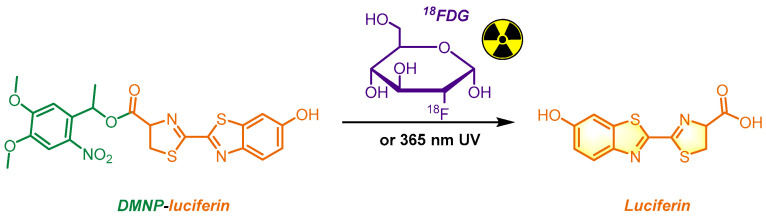
Luciferin release strategy utilizing Cherenkov luminescence generated by ^18^FDG.

Positron emission tomography (PET), a widely used imaging modality for tumor detection, identifies tumors within the body by detecting contrast agents containing radionuclides (such as ^18^F, ^64^Cu, ^68^Ga, etc.) that emit positrons (β^+^) [62]. During β^+^ decay, if the initial velocity of high-energy β^+^ particles exceed the speed of light in tissue, Cherenkov radiation is produced. ^18^F, with a half-life of 109.8 min, is an ideal radioisotope for PET, and 2-deoxy-2-[^18^F] fluoro-D-glucose (^18^FDG) is the most commonly used PET tracer in clinical practice [63,64]. In 2012, Ran et al. used ^18^FDG, a PET radiotracer, and photocaged luciferin to verify its ability to generate Cherenkov radiation and release free luciferin (Figure 9) [65]. In their in vivo experiment, the fluorescence signal intensity of luciferin 1-(4,5-dimethoxy-2-nitrophenyl) ethyl ester (DMNP-luciferin) combined with ^18^FDG was 12.5 times higher than that of ^18^FDG alone, peaking at 70 min after administration. DMNP, a well-known photocage unlocked by UV light at 365 nm, demonstrated high stability [66], offering strong evidence that ^18^FDG can generate Cherenkov radiation in vivo. Eleven years later, Guo et al. applied ^18^FDG to CRET-induced photodynamic therapy (PDT), utilizing goat milk-derived extracellular vesicles loaded with ^18^FDG and Ce6 to achieve CRET-induced PDT [67]. This photosensitizing system, including ^18^FDG, holds potential for therapeutic applications while simultaneously enabling PET imaging, paving the way for the integration of therapy and imaging.

**Figure 10:**
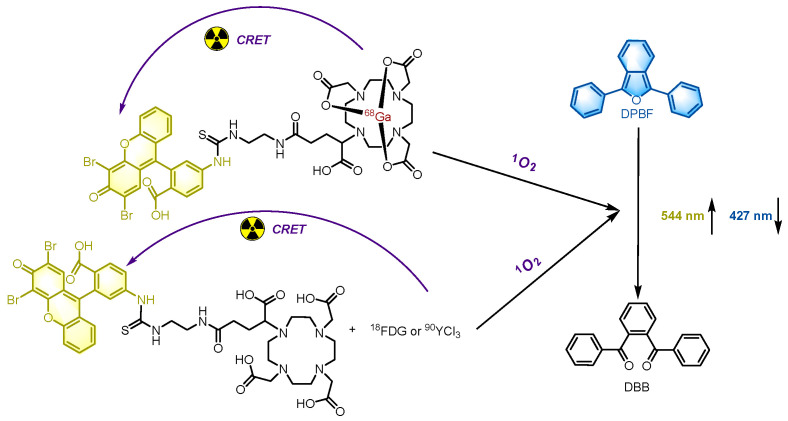
Strategy for combing eosin and ^68^Ga, ^90^Y, and ^18^F to achieve CRET-induced PDT.

The intensity of Cherenkov luminescence mainly depends on the selection of radionuclides [57]. Among them, ^82^Rb is considered the optimal, producing approximately 80 photons per decay. Other radionuclides, such as ^68^Ga, ^62^Cu, and ^90^Y, can also generate over 30 photons per decay, significantly outperforming the 1.3 photons per decay produced by ^18^F [64]. This disparity may explain why, despite the widespread clinical use of ^18^FDG, its combination with PDT is not particularly effective. In a study by Lioret et al., eosin (a type II PS, λ_abs_ at 524 nm, λ_em_ at 544 nm, and a fluorescence quantum yield of 0.67) was incorporated into the system to achieve PDT induced by Cherenkov luminescence from ^68^Ga, ^90^Y, and ^18^F [61]. They employed two approaches: directly mixing ^18^F-FDG or ^90^Y-YCl_3_ with eosin to achieve intermolecular-CRET or covalently binding ^68^Ga with eosin to facilitate both intra- and intermolecular CRET (Figure 10). The 1,3-diphenylisobenzofuran (DPBF), a popular ^1^O_2_ probe, was used to verify the PDT effect of these systems. Notably, eosin with and without ^68^Ga are essentially two distinct compounds, and their biological activities cannot be directly compared. However, ^68^Ga was found to significantly enhance the PDT effect. In cell experiments, when eosin was used at low doses where its inherent toxicity was negligible, its combination with radionuclides exhibited significant cytotoxicity, which can be attributed to PDT. As the exposure time increases, the cytotoxicity also escalated, likely due to the synergistic effects of radiotherapy, PDT, and the compound itself.

In 2023, Zhang et al. developed a hydrogel complex containing R837 (the immune adjuvant imiquimod) and ^89^Zr-HG-PpIX to achieve CRET-induced PDT [68]. This hydrogel exhibits minimal phototoxicity in normal tissues and low radiation leakage, with its safety validated in both in vivo and in vitro tests. Additionally, the overexpression of tumor-associated antigens induced by PDT, in combination with the adjuvant R837, significantly enhances the immune response in vivo. This advancement provides a solid foundation for the integration of CRET-induced PDT and immunotherapy, offering new possibilities for enhanced treatment.

CRET-induced PDT shares similarities with the combination therapy that integrates radiotherapy and PDT. In fact, several treatment approaches combining X-ray with PDT have also been developed based on radiotherapy [69,70]. However, this article focuses exclusively on the laser-free PDT systems, which are designed as self-exciting systems, independent of external triggers. Since X-ray-activated PDT has not fully surpassed this limitation, it will not be discussed in detail here.

## 5. Persistent Luminescence (PL) Systems

In recent years, persistent luminescence (PL, also known as afterglow luminescence) has emerged as a prominent research focus. Despite the fact that the PL systems requires pre-charging via UV irradiation in vitro and have not yet fully overcome the constraints posed by lasers, their ‘endurance’ is remarkably prolonged, lasting for several hours or even days, making them well suited for PDT applications [71]. While this review primarily focuses on laser-free PDT systems, it is evident that PL systems are steadily gaining significance in PDT research. The mechanism of PL luminescence is relatively intricate but can be summarized as the capture and storage of energy in traps through charge carriers (electrons and/or holes), followed by its gradual release facilitated by thermal energy. Based on this principle, Zhao et al. developed a DNA nanocomplex integrating persistent-luminescence nanoparticles (PLNPs) [72]. The surface of a PLNP is coated with a layer of MnO_2_, which helps reduce energy dissipation before reaching the tumor and responds to high expression of glutathione (GSH) in tumor tissue by generating oxygen to alleviate hypoxia in the tumor. Additionally, an AS1411 aptamer-conjugated ultra-long single-stranded DNA chain is incorporated to target the overexpressed nucleolin on tumor cells. This DNA chain also serves as a scaffold for the photosensitizer silicon phthalocyanine dichloride. In in vivo experiments, this strategy demonstrated an inhibition rate of over 80%, proving its effectiveness. However, the aptamer itself exhibited notable cytotoxicity, suggesting that further advancements are needed to develop novel, safe PL systems with a higher energy storage capacity and quantum yield. In the same year, Zhao published another study, incorporating 1-methyltrypsin alongside the aforementioned components. This modification enhanced T cell proliferation within tumor tissue, ultimately leading to the remodeling of the hypoxic and immunosuppressive tumor microenvironment (TME) [73].

Persistent luminescence (PL) systems face a significant challenge: energy dissipation in PLNPs after excitation. Even though certain PLNPs can sustain luminescence for several days, their energy gradually diminishes over time. Consequently, when PLNPs are introduced into the body, they exhibit their peak energy, maximum PDT activity, and the initial highest cytotoxicity. After several cycles and upon reaching the tumor tissue, their activity is significantly reduced. This intrinsic characteristic of PLNP cannot be addressed merely by structural modifications, which presents a considerable challenge in the development of the novel PL systems.

## 6. Limitations and Prospects

Photodynamic therapy (PDT) is a promising tumor treatment approach, but its effectiveness is often limited by the insufficient penetration of light through tissues. Recent advancements of photosensitizers (PSs) have gradually shifted from those activated by blue light to those activated by near-infrared light or even ultrasound. The advent of laser-free PDT systems has expanded the potential of PDT. Unlike traditional PDT, which relies on an external light source for photon activation, laser-free systems leverage energy transfer mechanisms such as RET (Resonance Energy Transfer) or CRET (Cherenkov Radiation Energy Transfer), as these mechanisms offer higher energy utilization efficiency and quantum yields. The key laser-free photodynamic systems discussed in this review, which are based on these advanced energy transfer mechanisms, are summarized in Table 1, highlighting their unique characteristics and potential applications. Additionally, laser-free systems typically generate rapidly decaying blue light from energy donors within the tissue, minimizing the risk to the surrounding normal tissues. This innovation has revived interest in PSs previously considered unsuitable due to their reliance on blue light activation, unlocking their potential for tumor therapy. Moreover, laser-free PDT systems can be seamlessly integrated with other anti-tumor therapies and are often compatible with treatment imaging modalities, making them highly valuable for enhancing the precision of personalized treatments.

Research on laser-free PDT systems is still in its early stages, and several challenges remain, preventing these systems from advancing to clinical trials. One of the most significant issues is their limited therapeutic efficacy. This limitation arises from the inherent constraints of the laser-free systems but also reflects a broader challenge in PDT, especially in the absence of external laser irradiation. While these systems, with their relatively weaker therapeutic capabilities, hold promise for integration with other cancer therapies, it is crucial to optimize the photosensitizing efficiency of PSs to maximize ROS generation. Achieving this optimization is essential to enhance the overall effectiveness of laser-free PDT and advance its potential as a viable treatment option.

In the RET process, the distance between the donor and acceptor must be smaller than the critical radius for effective energy transfer. If the molecules are not covalently linked, ensuring effective energy transfer could be challenging, and simply encapsulating them within nanoparticles may not adequately address this issue. Additionally, proper alignment of the HOMO energy level of the donor and the LUMO energy level of the acceptor in RET is also crucial, as improper alignment can significantly impede the energy transfer. In CRET systems, selecting an appropriate radionuclide is equally essential, requiring a careful balance of factors such as half-life, photon yield, and cytotoxicity to optimize performance and safety.

Furthermore, safety concerns also pose a significant challenge. Achieving self-excitation in many systems mentioned above requires administration of more than three components. This added complexity raises questions about the practicality of implementing such treatments. While some individual components may exhibit minimal cytotoxicity, their combined use in a more complex system could pose unforeseen risks to normal tissues. Additionally, some studies have failed to conclusively demonstrate that the observed anti-tumor effects are exclusively attributable to PDT, as opposed to the inherent toxicity of the compounds used. These studies often only report increased ROS levels, which makes certain laser-free systems diverge from traditional PDT, at times resembling chemotherapy or radiotherapy under the guise of PDT.

## 7. Conclusions

Due to the insufficient tissue penetration of light, photodynamic therapy (PDT) has been largely restricted to the treatment of superficial tumors, significantly hindering its clinical applications. The emergence of laser-free PDT systems may mark a potential turning point in the evolution of PDT. This article provides a comprehensive review of the latest advancements in laser-free PDT systems, highlighting key energy transfer principles in their design, including resonance energy transfer (RET), chemically induced electron exchange luminescence (CIEEL), and Cherenkov radiation energy transfer (CRET). Although still in its nascent stages, this field holds immense potential to expand the scope of PDT applications. This review aims to provide a theoretical framework to guide the development of novel laser-free PDT systems and to propose directions for future research in PDT and its integration with combination therapy for tumor treatment.

## Figures and Tables

**Table 1:** A summary of the key laser-free photodynamic systems discussed in this review.

Mechanism	Exciting Components	Photosensitizers	Other Components in the Nanosystem	Cytotoxicity In Vitro		Bioactivity In Vivo	Novelty	Ref.
RET	Luminol	5-aminolevulinic acid (5-ALA)	FeSO_4_	Cell viability ≈ 50% (Caco-2) with 3 mM 5-ALA, 0.2 mM luminol, and 0.1 mM FeSO_4_		-	The first in situ PDT mediated by luminol	[23]
	Luminol	Ce6	MPO	IC_50_ = 498.8 mM (RAW264.7), 414.2 mM (B16F10), 267.9 mM (MOVAS), 153.0 mM (MCF-7), 123.7 mM (A549)		Nanoparticle treatment (3.25 mg/kg of Ce6) group exhibited significantly reduced tumor size and weight	Weaker anti-tumor activity, providing a strong example of luminol-mediated chemiluminescence application in PDT	[25]
	Luminol	BODIPY (Compound **1** and **2**)	-	Cell viability < 60% (Hep-2) with 10 μM of drugs		-	The distance between BODIPY and luminol significantly affects quantum yield	[28]
	Luminol	Ce6	Paclitaxel (PTX) prodrug	IC_50_ = 0.37 μg/mL (with additional H_2_O_2_) and 2.1 μg/mL (without additional H_2_O_2_)		Marked reduction in tumor volume after combination therapy	Combination of laser-free PDT and chemotherapy	[27]
	Luminol	Ce6	Hybrid protein oxygen nanocarrier (HPOC)	Significant cytotoxicity on HeLa cells and primary cervical cancer cells after treatment with 16 μg/mL of nanodrug.		-	Hypoxia compensation, and immunogenic cell death (ICD)	[29]
	Luminol	IrL2	Hemoglobin	-		Tumor growth inhabitation, and non-toxic to normal tissues with 12 mg/kg of IrL2H.	Type I photosensitizer employed to reduce the dependence of laser-free PDT on tissue oxygen content	[30]
	e-coelenterazine	Ce6	*Renilla renormalizis* Luciferase 8 (RLuc8)	IC_50_ = 5.56 μM (4T1) and 1.51 μM (MDA-MB-231)		Complete eradication of early tumors in mice without metastasis	80% photosensitizer activation rate	[37]
	Furimazine	miniSOG	NanoLuc, riboflavin mononucleotide	Cytotoxic effect = 71% (BT-474 cells) with 75 μM furimazine		67% tumor-growth inhibition (TGI) coefficient	A completely genetical-encoded, laser-free PDT platform	[38]
	Furimazine	SOPP3	NanoLuc, DARPin_9-29	IC_50_ = 262.2 nM (BT-474, HER2^+^) and no significant cytotoxicity to Hela cells (HER2^−^)		Good targeting properties, 78% TGI coefficient	Improved treatment accuracy and reduced normal tissue damage	[39]
	Coelenterazine	Foscan^®^ (m-THPC)	RLuc8, QD-655	Cytotoxic effect = 50% (A549 cells) with 20 μg/mL coelenterazine		Significantly reduced tumor proliferation rate and lowered tumor tissue microvascular density	QD-655 is introduced as an intermediate to connect the donor emission wavelength and the acceptor excitation wavelength, expanding the selectable range of donor-acceptor pairs.	[41]
CIEEL	CPPO	Ce6	Glucose oxidase, perfluorohexane	Cell viability = 21% (B16-F10) with 0.2 mg/mL nanoparticles		Lung metastatic tumors were completely cured with PDT and starvation therapy	GOx catalyzes the conversion of intracellular glucose for synergetic starvation therapy, while the resulting H_2_O_2_ reacts with CPPO to enhance the PDT.	[48]
	CPPO	PpIX	HACP	Cell viability ≈ 50% (Hela cells) with 0.1 mg/mL nanoparticle and 5 μg/mL PpIX.		Most tumor areas showed necrosis with hemorrhage after treatment.	The supramolecular systems with large steric hindrance and enhanced stacking distance of photosensitizers show improved PDT efficacy. CA was introduced to activate NADPH oxidase in cancer cells and provide sufficient H_2_O_2_.	[50]
	Luminol	NIR-II dye D, Ce6	MPO	IC_50_ < 200 μg/mL (A549, L02, and MC38 cells)		This nanoparticle, as a therapeutic imaging integrated probe, can damage tumor blood vessels in vivo, and its luminescence signal is directly related to tumor volume.	The thiophene unit on dye D can be oxidized by ^1^O_2_ generated by RET activation of Ce6, resulting in NIR-II luminescence.	[52]
	CPPO	TPP	PFPV, folate	Cell viability = 33.5% with 40 μg/mL PFPV and 0.2 μM H_2_O_2_		The growth rate of tumors has significantly slowed down, and the weight of tumors has decreased by 85%	Addition of folic acid to significantly enhance the targeting and in vivo activity of PDT nanoparticles	[74]
	CPPO	Hypocrellin B	-	IC_50_ < 25 nM in A549 with ultrasound and 1 mM H_2_O_2_		-	An ultrasound-enhanced chemiluminescence PDT platform	[55]
CRET	^18^FDG	DMNP-Luciferin	-	-		-	^18^FDG, a commonly used PET probe in clinical tumor detection, is employed as Cherenkov light sources to break chemical bond and release luciferin.	[65]
	^18^FDG/^68^Ga	Eosin	-	^18^FDG	Cell viability < 50% (B16-F10) with 1 mM eosin	-	CRET mediated PDT with eosin as a photosensitizer to improve in vitro anti-tumor activity	[61]
				^68^GaCl_3_	Cell viability < 50% (B16-F10) with 0.5 mM drug (^68^Ga intermolecular) or 8 MBq ^68^GaCl_3_ (intramolecular)			
	^89^Zr	PpIX	R837 (immune adjuvant imiquimod)	Cell viability < 50% (H22 cells) with 10 μCi ^89^Zr		Strong immune activation and tumor elimination	CRET mediated PDT with adjuvants for a new combination therapy approach	[68]
PL	UV pre-excited nanoparticles (PLNP)	Phthalocyanine	MnO_2_, aptamer AS1411	-		80% tumor suppression rates in MCF-7-tumor-bearing mice	An oxygen-self-sustaining and self-irradiating persistent luminescence (PL) system	[72]

## Abbreviations

PDT	Photodynamic therapy
ROS	Reactive oxygen species
^1^O_2_	Singlet oxygen
PS	Photosensitizer
NP	Nanoparticle
RET	Resonance energy transfer
CL	Chemiluminescence
BL	Bioluminescence
CRET	Cherenkov radiation energy transfer
CIEEL	Chemically initiated electron exchange luminescence
TME	Tumor microenvironment
Ce6	Chlorin e6
PO	Peroxyoxalate
HOMO	Highest occupied molecular orbital
LUMO	Lowest Unoccupied Molecular Orbital
NIR	Near-infrared light
PpIX	Protoporphyrin IX
UV	Ultraviolet
UCEL	Ultrasound-enhanced chemiluminescence
HEI	High-energy intermediate
CTZ	Coelenterazine
PL	persistent luminescence
